# Between living and nonliving: Young children’s animacy judgments and reasoning about humanoid robots

**DOI:** 10.1371/journal.pone.0216869

**Published:** 2019-06-28

**Authors:** Minkyung Kim, Soonhyung Yi, Donghun Lee

**Affiliations:** 1 Department of Child Development and Family Studies, College of Human Ecology, Seoul National University, Seoul, Korea; 2 School of Mechanical Engineering, Soongsil University, Seoul, Korea; University of Montana, UNITED STATES

## Abstract

Humanoid robots will become part of our everyday lives. They have biologically inspired features and psychologically complex properties. How will children interpret these ambiguous objects, discriminating between living and nonliving kinds? Do the biologically and psychologically inspired characteristics affect children’s understanding of the robots? How firm is the distinction that children make between living and nonliving objects? To address these questions, 120 children ranging three to five years initially viewed video clips that depicted humanoid robots interacting with a human experimenter on two different dimensions (mobility and, psychologically contingent behavior). The subjects then answered simple questions that probed their animacy judgments and property projections about the robot. The results showed that children’s animacy assessments about humanoid robots differed by age. When judging the robot’s life status, its mobility was important for four-year-olds and, the psychological contingency for five-year-olds. In terms of the robot’s reasoning abilities, the majority of four-year-old children clearly understood biological properties, regardless of the robots’ features. However, when reasoning about psychological properties, even five-year-olds occasionally relied on robots’ features such as their contingent behaviors. Moreover, the children attributed some but not all animate properties to the robots. Although rent findings show that children possess naïve theories, they do not seem to have a consistent and logical theory of “aliveness,” and they apparently develop the concept of a robot by acquiring knowledge about how this boundary object differs from living entities.

## Introduction

Many researchers have long been interested in young children’s biological concepts. Despite much exciting work on children’s conceptions of the biological world, numerous debates continue on this topic. From the perspective of nativism, even infants are considered to have the ability of representing biological concepts (e.g., [1–4]) which nativists assume to be innate and their structure qualitatively the same throughout infancy, early childhood, and school age. In the theory-theory of concepts, children are regarded as “theory builders” with consistent and logical theories. Some researchers proposing naïve biology (e.g., [5]) have reported that young children can understand the biological processes shared by living entities and distinguish between living and nonliving kinds.

In contrast, Piaget [6], the most representative constructivist, argued that the concepts in infancy, early childhood, and school age are qualitatively different. He explained that children at the preoperational stage think in an animistic manner that hinders them to distinguish between living and nonliving objects [6]. Although the beginning of concept development in Carey’s research [7] is earlier than Piaget’s [6], she explained—in the same vein—that the conceptual structure is continually restructured in the cognitive development of children. In addition, she mentioned that young children’s understanding of biology is inaccurate because their pertinent knowledge stems from a previous understanding of psychology [7].

To date, there have been some limitations in the literature on children’s biological concept development. In many studies, researchers examined children’s understanding about objects that were clearly classifiable as living (e.g., animals) or nonliving (e.g., hand tools). It is not surprising that they could distinguish these simple everyday items. Despite the plethora of work on this topic, there are few studies about potentially confusing objects at the borderline between the animate and inanimate. In order to extend the current work on children’s understanding of living-nonliving distinctions, it is necessary to examine how they grasp items intellectively with different animate properties.

Robots obscure traditional ontological categories while becoming more popular and familiar in our everyday lives, which is why studies [8, 9] related to children’s distinction between living and nonliving kinds have resumed. Robots such as Pepper from Aldebaran or Jibo from MIT can socially interact with humans and are quite lifelike. Identifying these boundary objects can be a challenge to young children since they cause conflicts with their existing categorization mechanisms. By investigating how children negotiate such conflicts and how they reason about the boundary objects, we can identify features that might be relevant to children’s understanding of animacy.

Two important attributes that have been mentioned in the literature as influences of children’s category judgments are mobility and contingent reactivity. Children treat object differently, depending on its autonomous movement [10–12]. For example, children reported natural objects like moving clouds as living entities in Piaget’s studies [6]. Carey demonstrated that young children could not group plants together with animals into the category of living kinds because they did not perceive plants’ movements [7]. Such findings suggest that the mobility of objects may affect children’s thinking about the objects’ animacy. However, it is unclear whether this feature is equally important for children of different ages. If mobility affects the children’s living-nonliving distinction, their reasoning may be different, depending on the experimental manipulation of object mobility. However, if they possess biological “*theories*” like adults, an object’s perceptual features such as mobility would not affect their reasoning about the living-nonliving distinction. An objects’ contingent response is one of the other attributes that have received attention. Robots designed to interact socially seem able to respond contingently to humans. For example, they sometimes express emotions and behave like intelligent agents with cognitive ability. These features distinguish existing artifacts from intelligent robots. Turkle described robots as "relational artifacts" and their psychological features are powerful elements that blur the living-nonliving boundaries [13]. Researchers have manipulated robot’s contingency so that it could engage in socially communicative exchanges with an adult experimenter. Even infants use information derived from a robot’s social-communicative interaction with an adult as evidence about whether that the robot is a psychological agent [14]. Preschoolers were sensitive to the social responsiveness of robot and displayed a preference for the contingent robot as an informant [15]. This result suggests that the contingent responsiveness of the robot is likely to be one important contributor to such receptivity and may affect children’s understanding of robot.

Therefore, in this study, we examined whether children draw different inferences about humanoid robots with various features by manipulating mobility and contingent reactivity. From the perspectives of nativism and constructivism, we see two explanations about children’s reasoning regarding boundary objects such as robots. One hypothesis states that children’s biological concepts constitute a logical and scientific “*theory*” and the essential properties of the living entities should be an important basis for the living-nonliving distinction. An alternative hypothesis posits that children’s biological concepts are illogical and intuitive: Their reasoning may be affected by the perceptual features of robots rather than by considering the essential properties of living things. In short, we aimed at verifying whether children’s understanding of the animacy concept is consistent and logical through the examination of their reasoning about different robot types.

Previous studies on children’s reasoning of animacy are limited in their focus on specific parts of biological properties. The generalization of these individual research results into biological knowledge [16] is restricted, because it cannot be confirmed that the child has inferred both the biological and the psychological phenomena in the same pattern. Children may distinguish categories of items based on specific properties included in a single domain, such as the biological one, or they may classify items through various features such as in the biological and psychological domains. For example, a child may know that an object is not alive but s/he may attribute psychological properties such as emotions or cognitive ability to it. Therefore, it is necessary to take children's reasoning about various domain-specific properties into account for understanding their biological concepts. This allows determining whether children infer animacy in a single or in a more multifaceted way.

Recent studies on children’s understanding of boundary objects have mostly used animal robots as experimental stimuli (e.g., [17–19]). Although previous research demonstrated children’s understanding about boundary objects with animal features, their comprehension of corresponding objects similar to humans may be different. Studies with induction tasks demonstrated that children deduce in a way that resembles analogies between subjects and humans. There are also findings about children’s inductive reasoning based on the physical similarity of objects (e.g., [20]). This tendency can be extended to beings with physical characteristics similar to humans [21] which suggests that children may infer differently between humanoid robots and animal robots. Since the former have similar appearances and comparable behaviors as humans, the possibility exists that children face strong conflict situations when asked to classify humanoid robots into living and nonliving categories. Unless their biological concept is different from adults, nativism would assert that children consistently classify humanoid robots as nonliving artifacts. On the other hand, if biological concepts are unstable because they continuously develop in early childhood, children may experience confusion in animacy reasoning about humanoid robots. Therefore, it is necessary to examine their judgment and reasoning about humanoid robots which is conceivably one of the most challenging tasks for children in performing living and nonliving categorizations among boundary objects.

This study extended previous work on children’s biological concepts in several ways, especially the distinction between living and nonliving kinds. First, we examined specific features that may be relevant to children’s category living-nonliving judgments. Using humanoid robot can be an effective method which features have a significant influence on children’s biological concepts because those features can be experimentally manipulated. By systematically varying a set of features that may affect the categorization judgment of children, it can be determined whether a robot moves autonomously and whether it behaves in a psychologically contingent way. Second, we explored whether children include a single categorical distinction between domains in animacy judgments or whether they infer living and nonliving things more extensively. To this end, we investigated children’s reasoning about biological and psychological properties about robots. Finally, we examined the influence of reasoning about a robot’s properties on children’s animacy judgments. In this way, the developmental patterns of children’s biological concepts may be identified and the theoretical debates on nativism and constructivism about this topic clarified.

## Methods

### Participants

One hundred twenty preschool-aged children participated in this study, including forty three-year-olds (*M =* 43.75 months, range = 38–47 months, 22 girls), forty four-year-olds (*M =* 55.80 months, range = 50–59 months, 19 girls), and forty five-year-olds (*M =* 67.48 months, range = 61–71 months, 21 girls). The participants were all Korean and from middle-income homes. Two three-year-olds were excluded from the analysis because they failed to finish the study. The participants were recruited from two daycare centers and one kindergarten in Seoul and Gyeonggi province, South Korea. The reason for choosing the age of the study subjects was that there has been controversy as to whether naïve biology exists in children at the preoperational stage [5–7, 22]. In addition, the naïve biology of preschool-aged children without formal biology education could be examined. To control for the influence of experience about intelligent robots on task performance, children with prior experience of humanoid or animal robots were excluded, as determined through parent questionnaires. Written informed consent was obtained from the parents of the subjects. The study was approved by the IRB of Seoul National University.

### Stimuli

A new intelligent humanoid robot named Vex (Various facial expression robot), was built to study the development of children’s biological concepts and employed as a stimulus for the experiment. The reason for using a “humanoid” robot as the target stimulus laid in the control of the influence that appearance can bear on children’s judgments and reasoning about robots [17]. Vex is a medium-sized humanoid robot, 530 mm (H)× 220 mm (W) × 150 mm (D), made of plastic and metal, and designed to be attractive for children [23]. Vex has a face, eyes, arms, legs, and a torso. It is able to interact with children through motions, facial expressions, and speech. Vex has been designed to implement various facial expressions. It allows 17 degrees of freedom, including two degrees of the neck, hip, and of the ankle each, as well as one degree each of the shoulder, the elbow, the waist, and the knee.

Participants viewed four 30–90 s video clips depicting a female experimenter interacting with Vex under four different experimental conditions: (R1) immobile and non-contingent, (R2) immobile and contingent, (R3) mobile and non-contingent, and (R4) mobile and contingent. Those experimental conditions varied on the following dimensions: autonomous movement and psychologically contingent behavior of the robot. All video clips were displayed on a laptop computer.

### Design

#### Experimental conditions of the humanoid robot according to its features

**Fig 1:**
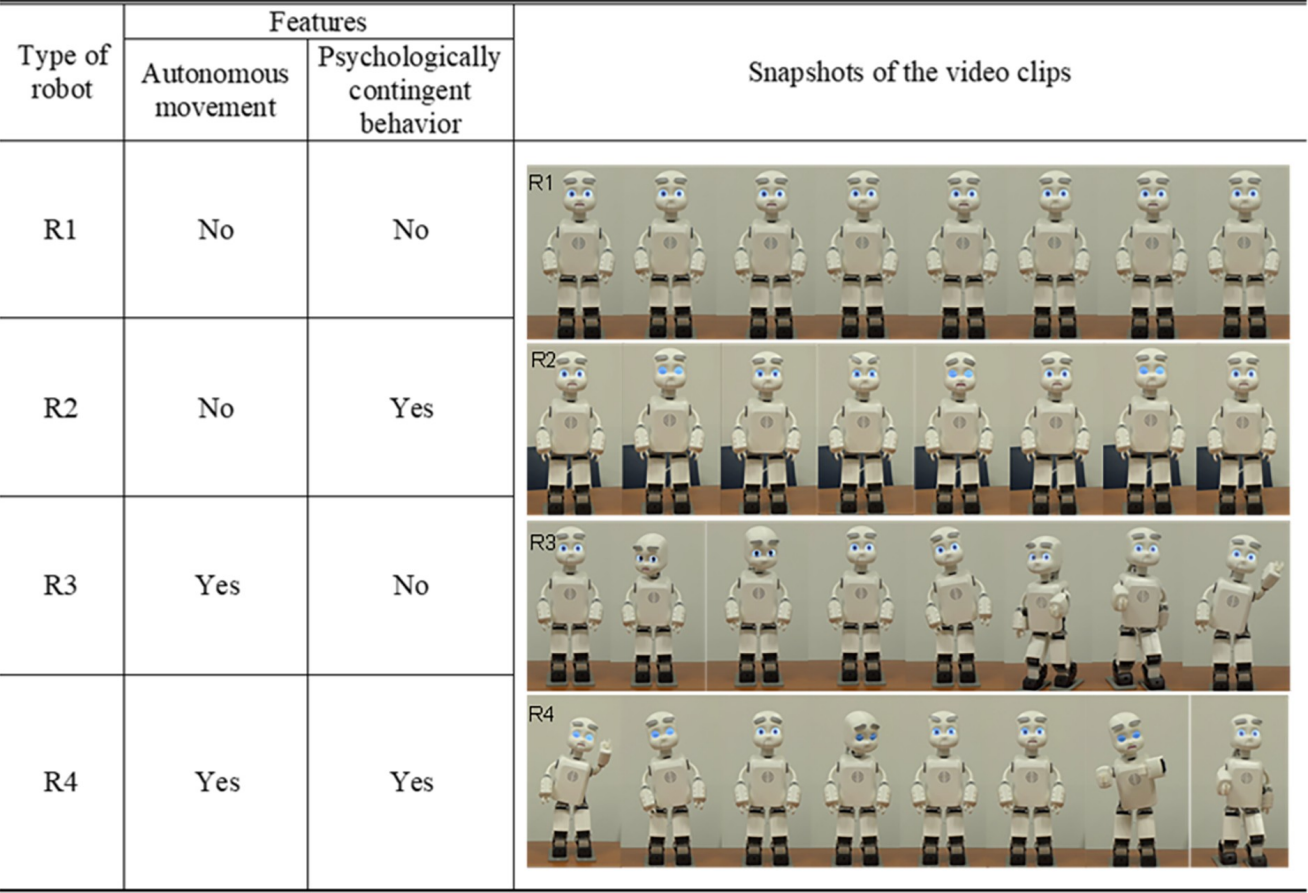
Four types of robots with their associated features and snapshot examples of the video clips.

Vex was programmed so that it could be classified into four types depending on its autonomous movement and psychologically contingent behaviors (Fig 1). In the immobile and non-contingent condition (R1), the robot stood still, stared at the front and continuously made no facial expression. In the immobile and contingent condition (R2), Vex did not move while gazing at the front, but showed socially acceptable behavior such as appropriate facial expressions depending on the experimenter’s cues. In the mobile and non-contingent condition (R3), it moved its head, arms, and legs randomly without any emotional expression. In the mobile and contingent condition (R4), the robot responded as naturally and socially as possible to the experimenter by appropriate movements and facial expressions to the experimenter’s cues.

All types of robot had a speech function, but there were differences depending on the robot’s ability for psychological contingency. In the non-contingent conditions, the robots (R1, R3) produced mechanical speech without meaning. They emitted nonsense sounds every half second for 35 seconds (e.g., ne, chin, goo, hon, up, uh, an). In contrast, with psychological contingency, the robots (R2, R4) used socially appropriate speech with a humanlike voice depending on the experimenter’s cues. That is, these robots were able to engage in natural conversation with the human experimenter.

#### Experimental settings

The four interaction patterns (in italics) were designed as follow. First, *greeting*: The female experimenter came into the laboratory and met the robot. She greeted the robot and the robot greeted back (R2 and R4) or not (R1 and R3). In the second interaction pattern, *introducing oneself*, the experimenter asked the robot for a self-introduction and it responded differently, depending on the experimental condition. Psychologically contingent robots (R2, R4) introduced themselves with appropriate facial expressions and speech. However, non-contingent ones (R1, R3) displayed random actions in response to the experimenter’s self-introduction requests. For *showing off mobile ability*, the robot (R4) performed movements depending on the experimenter’s request. In *reciprocal compliments*, the robots (R2, R4) returned a compliment that they had received from the experimenter. Taken together, the robots’ behaviors differed, depending on the varied conditions, even though the experimenter’s behavioral initiative was the same in all experimental contexts.

#### Interview

The interview developed for this study consisted of a question about the living-nonliving distinction and a set of six property projection items. The questions were based on previous studies about children’s biological concept development [16–19, 24, 25]. The animacy judgment question between living and nonliving kinds required the children to judge whether or not each robot was alive (e.g., “Is it alive or not alive?”). After responding to the animacy judgment, the subjects were asked a set of six property projection questions from the following domains: biological (Eating: “Does it need water or food?,” Growing: “Does it grow?,” Breathing: “Does it breathe?,” Origins: “Was it born? Or was it made by man?”), and psychological (Emotion: “Can this one feel happy or unhappy?,” Thinking: “Can this one think?”). Cronbach’s alpha value of the biological property measure is .902 and the alpha value of the psychological property measure is .875.

### Procedure

All children were tested individually in an experimental space (150 × 100 cm) within a quiet room of each daycare center and kindergarten. At the space’s center, there was a laptop computer placed on a table where the researcher and the participant sat. A video-watching period was followed by an interview. The researcher presented a PowerPoint slide to the children that listed the photographs and names of the four robot types. Afterwards, each robot was introduced to the participants by four different names so that the children could distinguish the different types. The subjects then viewed all four video clips in the order of either ‘R1, R2, R3, and R4’, or ‘R1, R3, R2, and R4’. If a robot with more features was presented earlier, it could affect the child’s understanding of the one following it. Therefore, in the same way as a previous research [26], the robot with less features was shown before that with more. Following each video, the participants were asked to judge the animacy and then the six property projection questions set in random order. During the interview, the robot was referred to as “it” to ensure that the name did not affect the judgment and reasoning about the robot [17, 24]. The time required for each subject was about 15 minutes.

### Analysis

Statistical analyses were performed using SPSS Win 18.0. The methods used were chi-square tests, Fisher's exact tests, repeated-measures ANOVAs, F tests, and logistic regressions. To determine whether the children’s judgments differed by age, a chi-square test was performed for each type of robot. The reasoning score was used to assess the extent to which children attributed biological and psychological properties to the robots. Then, repeated-measures ANOVAs determined whether the reasoning scores were significantly different according to the children’s age and the robot types. We also analyzed children’s reasoning for sub-items of the biological and psychological properties to explore the concrete reasoning about the robot’s life phenomena. Chi-squares and Fischer's exact tests examined whether the children's reasoning about the biological and psychological property sub-items significantly differed by age and the robot type. Finally, we conducted a binary logistic regression analysis to scrutinize the relative influence of children’s reasoning of biological and psychological properties on their understanding of the robots’ living-nonliving distinction.

## Results

The results are reported in three sections. The first one describes results from children’s animacy judgments about the humanoid robots. The second section reports participants’ responses to the property projection questions and the final section analyzes the relative influence of children’s property projections on their animacy judgment about the robot.

### Children’s understanding of the humanoid robots’ living-nonliving distinction

There were significant differences in the animacy judgments according to age (S1 Table) with the largest gap with regard to the robot (R1) in the immobile and non-contingent condition (χ^2^ = 21.06, df = 2, p < .001). As a result of standardized residuals, the percentage of 3-year-olds who answered that R1 is alive was significantly higher and the percentage of 5-year-olds who answered that R1 is alive was significantly lower. For the more specific age difference verification, we conducted a post-hoc analysis of the age-based partition table. As a result, there was a significant difference in the animacy judgement between 3-year-olds and 4-year-olds (χ^2^ = 5.23, df = 1, p < .05), between 4-year-olds and 5-year-olds (χ^2^ = 6.05, df = 1, p < .05), and between 3-year-olds and 5-year-olds (χ^2^ = 20.83, df = 1, p < .001). Comparing 3, 4, and 5-year-olds, the younger children responded more that R1 is alive. The second-largest age difference in the animacy judgment pertained to robot (R3) in the mobile and non-contingent condition (χ^2^ = 15.95, df = 2, p < .001). As a result of standardized residuals, the percentage of 3-year-olds who believe that R3 is alive was significantly higher and the percentage of 5-year-olds was significantly lower. The results of post-hoc analysis shows that there was a significant difference in the animacy judgement between 4-year-olds and 5-year-olds (χ^2^ = 6.37, df = 1, p < .05), and between 3-year-olds and 5-year-olds (χ^2^ = 14.59, df = 1, p < .001). Compared to five-year-olds, younger children were more likely to believe that R3 was alive. This result was consistent with earlier work where an object’s mobility was important for the understanding of animacy in three- to four-year-old children (e.g., [6, 10–12]). However, for five-year-olds, mobility did not appear to be important compared to younger children.

The third-largest age difference of animacy judgments was in R2, the immobile and contingent condition (χ^2^ = 12.29, df = 2, p < .01), accounted for by the gap between the ages of 3 and 4 (χ^2^ = 9.04, df = 1, p < .01). That is, as compared to four- or five-year-olds, younger children tended to believe that R2 was a living creature. Comparing the percentage of 4-year-olds’ reporting the robot is alive, there are more children who answered that R2 is alive than children who answered that R1 is alive. However, more children reported R3 to be alive than R2. Five-year-olds were more likely to believe that R2 was alive as compared to R1 or R3. Such findings suggest that—although mobility is an important criterion for four-year-olds’ animacy judgments—five-year old children seemed to judge the objects’ life status by linking psychological features to animacy.

**Fig 2:**
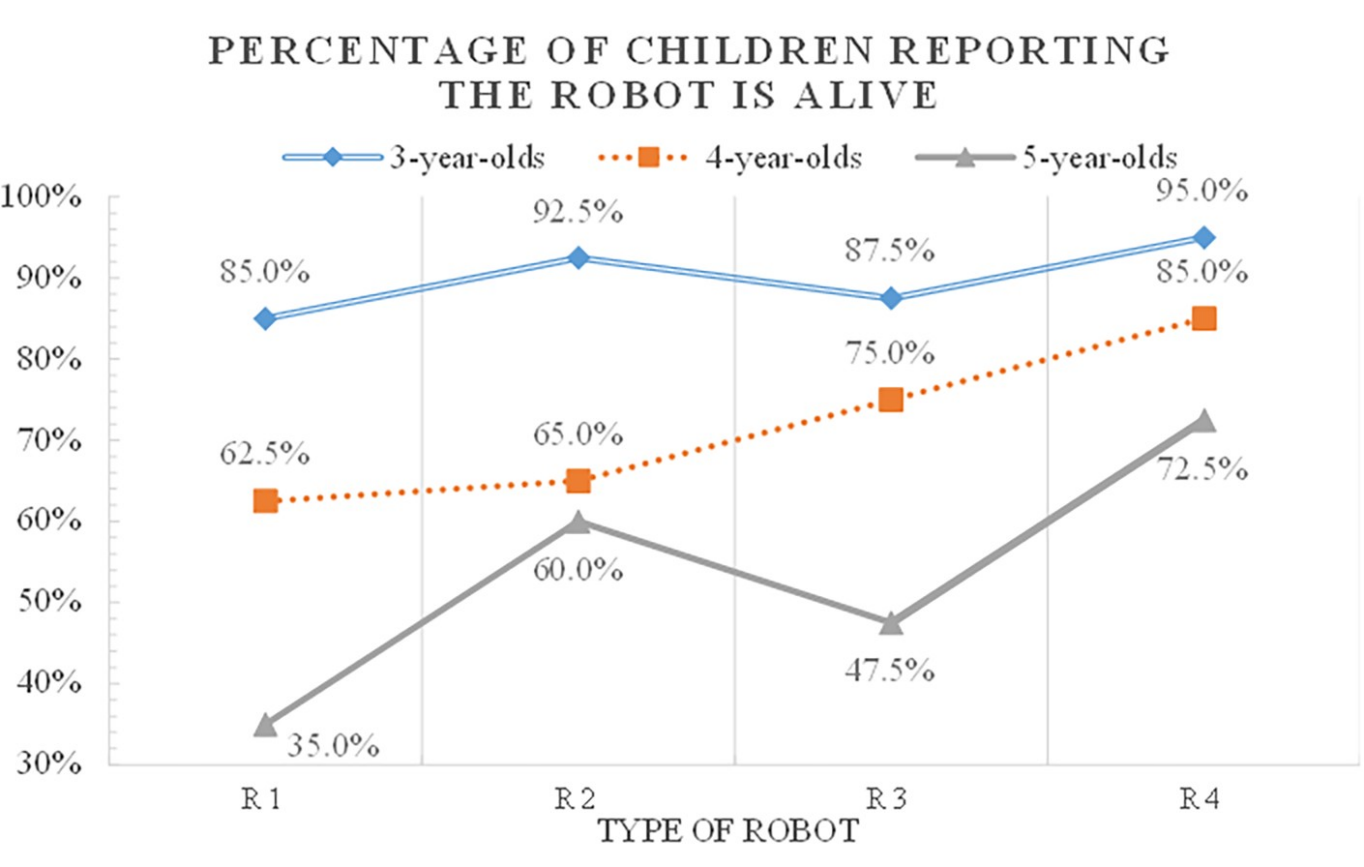
Proportion of children’s animacy judgments by age and robot types.

The smallest age difference in animacy judgments was in the mobile and contingent robot (R4) condition (χ^2^ = 7.63, df = 2, p < .05). More than 70% five-year-olds reported that R4 was alive. The results of post-hoc analysis shows that there was a significant difference in the animacy judgement only between 3-year-olds and 5-year-olds (χ^2^ = 7.44, df = 1, p < .01). Although older children were less likely to believe that humanoid robots were alive than younger ones, differences in animacy judgments according to robots’ features existed (Fig 2).

Five-year-olds’ animacy judgments were significantly different according to robot’s features (χ^2^ = 12.57, df = 3, p < .01). They believed that R2 and R4 were alive as compared to R1 (χ^2^ = 5.01, df = 1, p <. 05 and χ^2^ = 11.31, df = 1, p < .01, respectively). In addition, the number of five-year-olds who reported that R4 was alive was significantly higher than that of R3 (χ^2^ = 5.21, df = 1, p < .05). There were no significant differences in five-year-old children’s animacy judgments between robots with different mobility, e.g., five-year-olds’ R1-R3 or R2-R4 comparison judgments were not different. Hence, mobility was not an important feature for them and they were more sensitive to robots behaving in a psychologically contingent way. Both three-year-olds’ animacy judgments (χ^2^ = 2.78, df = 3, p = .427) and four-year-olds’ animacy judgments (χ^2^ = 6.28, df = 3, p = .099) did not differ significantly based on robot’s features.

In summary, factors affecting the prediction of children’s animacy judgments on humanoid robots varied by age. Three-year-olds tended to judge robots as living beings regardless of their features. In four-year-olds, mobility seemed to influence their animacy appraisals. This finding is consistent with earlier work on children’s animistic thinking [6, 10–12, 19]. Robots displaying psychological features like emotions seemed to affect five-year-old children’s animacy judgments: They were influenced by the boundary objects’ characteristics and had developed a better biological concept than younger children. Therefore, their animacy judgments were not strongly influenced by appearance and mobility. Rather, children at this developmental period may react sensitively to psychological features such as contingent interaction that have been used as important clues for their animacy judgments. Our results support the constructivist view of biological concept development that children’s living and nonliving distinctions are different according to the target objects.

### Children’s reasoning of biological and psychological properties of humanoid robots

The children’s responses to each of the property questions were scored as the number of “yes” responses given to the different question types (biological, psychological) and according to the robot types. The theoretical range for biological-property question scores was from 0 (did not attribute either property to the robot) to 4 (attributed all properties to the robot) and that for psychological property from 0 to 2.

#### Biological property projection on humanoid robots

To determine how children at each age reasoned about the properties of the robots, the scores were analyzed with a repeated-measures ANOVA (S2 and S3 Table). Age was the between-subjects factor and the robot type the within-subjects factor. Main effects were found for age and type of robot, F(2, 117) = 56.92, p < .001 and F(3, 298) = 11.36, p < .001, respectively. The interaction effect was not significant, F(6, 298) = .38, p < .001.

**Fig 3:**
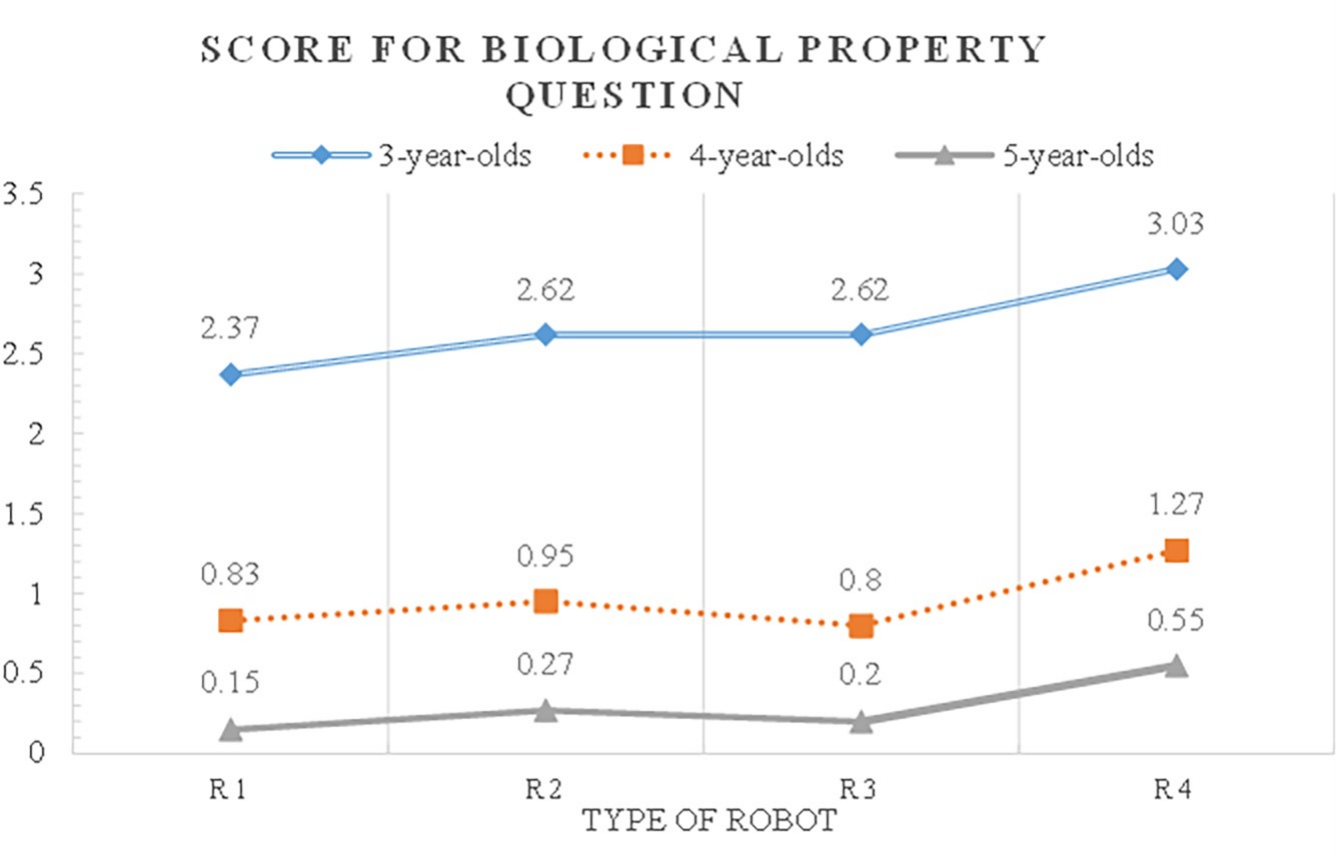
Children’s biological attribution scores for humanoid robots by age and types of robots.

The post-hoc test (Scheffé) was conducted to see the difference between the age groups. The children attributed more biological properties in the descending order of age 3, 4, and 5 (Fig 3) which indicates a developmental difference in the reasoning of biological properties about the robots. Specifically, three-year-olds attributed more biological properties than both four-year-olds (p = .000, d = 10.625) and five-year-olds (p = .000, d = 14.813). Four-year-olds attributed more biological properties than five-year-olds (p = .016, d = 4.187). Three-year-olds seemed to reason that the robots had many biological properties because of their perceptual characteristics. However, four- and five-year-olds appeared to understand that robots had inanimate properties.

The differences in biological property projection were significantly different, according to the number of the robots’ features (Helmert contrasts). Children attributed more biological properties to R2 (p = .000, d = 0.106) with contingency and to R3 (p = .000, d = 0.060) with mobility than R1 with no mobility and contingency. Moreover, children attributed more biological properties to R4, which possessed both contingency and mobility in contrast to the robots R2 (p = .102, d = 0.212) and R3 (p = .000, d = 0.256) that exhibited only contingency or mobility. These findings suggest that mobility and contingency are important factors when children reason about robots’ biological properties.

**Biological property projection of children who believe the robots are alive**

**Fig 4:**
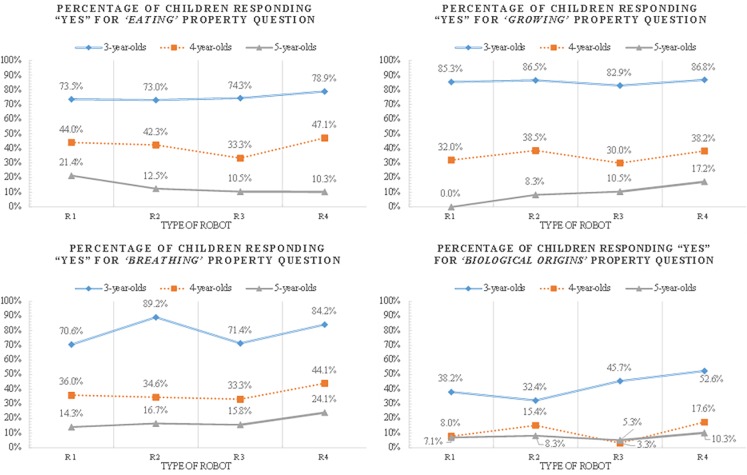
Proportion of children’s biological property projections by age and robot types.

Subordinate reasoning patterns for biological properties differed by age (S4 Table, Fig 4). Firstly, there was a developmental difference in reasoning about robots’ biological properties such as “eating,” “growing,” and “breathing”. Younger children reported more than older ones that robots ate food, drank water, grew, and breathed. Although robots imitated human-like characteristics, most five-year-olds did not attribute such features to robots and they seemed to understand that robots are artifacts without biological properties.

Secondly, there was a significant age difference in the “origins” property projection, but not according to the robot types. Three-year-olds were more likely to report that robots (R1, R3, and R4) were born biologically as compared to four- and five-year-olds who seemingly understood that robots are man-made artifacts. Interestingly, even children as young as three years of age assessed robots’ origins more accurately than other biological properties. In contrast to their inferences about eating, growing, and breathing properties, the majority of them reasoned that robots are man-made artifacts although other biological properties such as eating, growing, and breathing still existed.

These findings suggest that older children understand robots’ biological properties better than younger children, which is consistent with previous research that established the age of 4 as a transitional period in reasoning development [22]. In addition, the result that biological property projections did not differ according to the robot type can be interpreted in a way that children understand biological properties in a consistent fashion.

#### Psychological property projection on humanoid robots

A repeated-measures ANOVA was calculated to examine how children at each age reasoned about the robots’ properties (S5 and S6 Table). Age was the between-subjects factor and the type of robot the within-subjects factor. Main effects were found for age and robot type, F(2, 117) = 12.90, p < .001 and F(3, 323) = 36.19, p < .001, respectively. The two-way interactions were also significant at the p < .001 level: age × type of robot, F(6, 323) = 5.68, p < .001. In order to investigate the interaction effect, the simple main effect was analyzed though F test (S7 Table).

**Fig 5:**
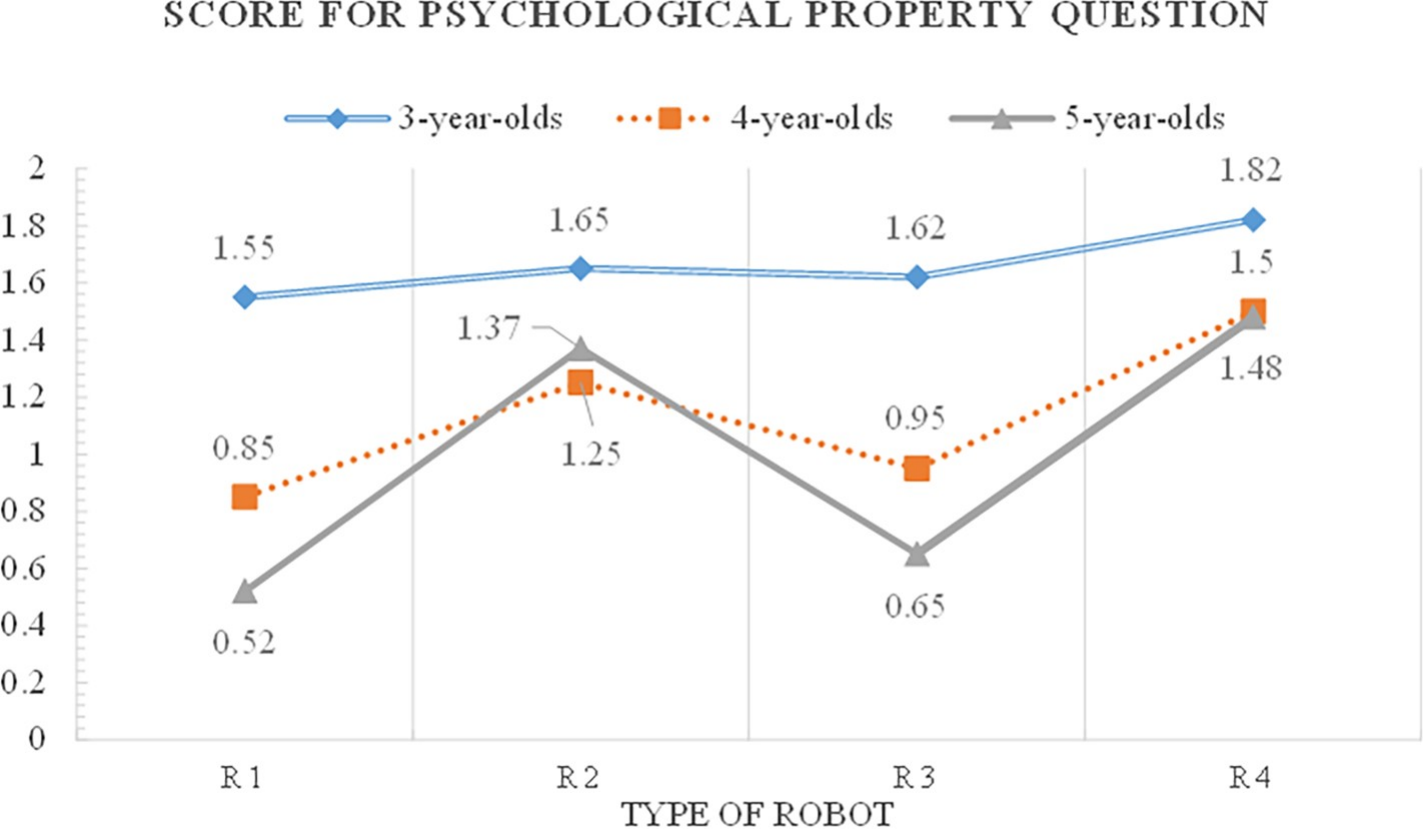
Children’s psychological attribution scores for humanoid robots by age and robot types.

Three-year-olds attributed more psychological properties to robots than both four-year-olds (p = .001, d = 5.20) and five-year-olds (p = .000, d = 6.50) (Fig 5). There were no significant differences in psychological properties between four- and five-year-olds (p = .632, d = 1.30). The children attributed psychological properties differently, depending on the robot type. More psychological properties were attributed to psychologically contingent robots (R2 and R4) than to non-contingent ones (R1 and R3). Specifically, children attributed more psychological properties to R4 which has mobility and psychological contingency than both R1 (p = .000, d = 0.772) and R3 (p = .000, d = 0.643). Children attributed more psychological properties to R2 which has psychological contingency than R1 (p = .000, d = 0.537). They also attributed more psychological properties to R3 which has mobility than R1 (p = .000, d = 0.109). On the other hand, there were no significant difference between psychologically contingent robots which are R2 and R4 (p = .140, d = 0.236) and between psychologically contingent robot R2 and mobile robot R3 (p = .140, d = 0.415).

There was a significant age by type of robot interaction effect (S7 Table). Three-year-olds tended to attribute psychological properties to all types of robots and they seemed to perceive humanoid robots as agents with minds, being “personified” by perceptual characteristics such as a human-like appearance, especially with a face and eyes [27], autonomous movements [27–29], and vocal abilities. By four years of age, children attributed psychological properties more to R4 than any other robots, F(3, 117) = 4.76, p < .01, implying that both psychological and biological features of robots are important to deduce that robots have emotions and thinking abilities. Five-year-olds significantly differed in their psychological property projections for all four types of robots, F(3, 117) = 16.43, p < .001. They attributed higher scores to robots in descending order of R4, R2, R3, and R1 that—again—is suggestive of psychological contingency as an important cue in psychological property projection. In particular, this can be interpreted as a reflection of the understanding how intentions and behavioral consequences relate, which stabilizes after age 4 [30] and requires the ability to comprehend the state of mind as a representation. Psychologically contingent robots (R2, R4) responded appropriately to the speech instructions and behaviors of the experimenter. Five-year-olds appeared to have recognized clearly that there are robots’ contingency differences. Humanoid robots that contingently interacted with the experimenter seemed to induce strong categorical conflict situations, even for five-year-olds and they affected the children’s understanding about the boundary objects as agents with minds [27], which leads to the psychological property attribution to robots.

Unlike three-year-olds, four- and five-year-olds seldom attributed biological properties to all types of robots. However, four- and five-year-olds’ psychological property projections were different according to the robots’ features. Such results suggest that children do in fact have a concept of a biological thing as something separate from a psychological thing. In other words, it seems that the aspect of children’s reasoning about biological properties and the reasoning about psychological properties were different.

**Psychological property projection of children who believes the robot is alive**

**Fig 6:**
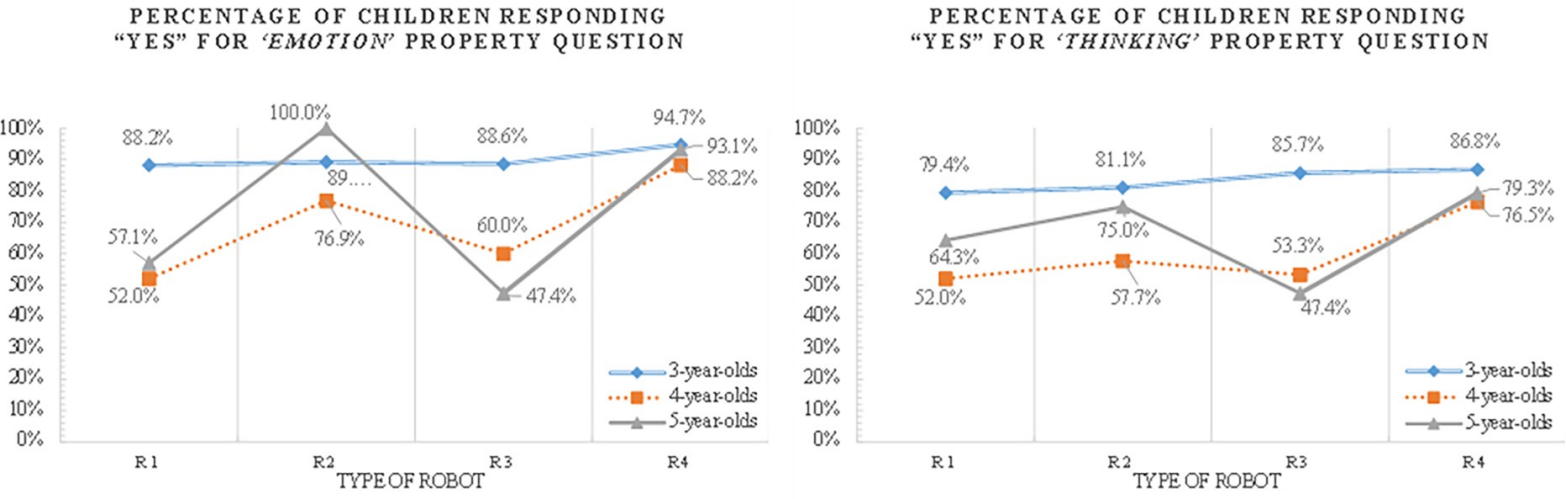
Proportion of children’s psychological property projection by age and robot types.

Subordinate reasoning patterns of children for psychological properties differed by age and the type of robot (S8 Table, Fig 6). There was a statistically significant age difference in the “emotion” property projection for R1 (χ^2^ = 10.32, df = 2, p < .01). Specifically, three-year-olds reported more than four- and five-year-olds that R1 can feel emotions. In the case of R3, there was a significant difference in “emotion” (χ^2^ = 11.57, df = 2, p < .01) and “thinking” (χ^2^ = 11.06, df = 2, p < .01) property projection between 3-year-olds and 4- to 5-year-olds. Three-year-olds may have used appearance [27, 31, 32] and mobility [27, 29] as a clue to infer that the robot possesses psychological properties while robots’ morphological characteristics or their autonomous mobility constituted an insufficient basis for older children’s attributions of psychological properties to robots. When the robot responded appropriately to the experimenter and expressed emotions with facial expressions, children might have perceived the robot as an agent with a mind [27] and deduced its psychological properties.

The difference in reasoning about robots’ “thinking” property was smaller compared to that about its “emotion.” Four- and five-year-olds seemed to be more affected by robots’ features when asked to reason about robots “emotion” as compared to “thinking” properties. This difference seems to be due to the fact that children used robots’ affective expressions as cues to determine the presence of “emotion” properties. Inferences regarding the presence of robots’ thinking abilities might be a more difficult task for children since they base their inferences on whether robots can “feel emotion” and because they had to identify a characteristic that was not readily visible on a surface level.

### Relative influence of children’s property projections on animacy judgments about humanoid robots

We also conducted a binary logistic regression analysis by age groups to examine whether the effects of biological and psychological property projections on children's animacy judgment were different by age (S9 Table). First, the prediction success rate of animacy judgments classification about humanoid robot for three-year-olds was 89.4% when their biological and psychological property projections were included in the model as independent variables. In addition, this model was statistically suitable for predicting three-year-olds’ animacy judgments about humanoid robots (χ^2^ = 16.62, df = 2, p < .001). At three years of age, the children’s biological property projection was found to have a significant effect on the prediction of their animacy judgments judgments (B = .50, p < .05). Specifically, if the biological property projection scores increased by one point, the likelihood that three-year-olds judged the robot to be alive increased by 1.65 times. The prediction success rate of animacy judgments classification about humanoid robot for four-year-olds and for five-year-olds were 71.9% and 76.3% when their biological and psychological property projections were included in the models as independent variables. In addition, these models were statistically suitable for predicting four-year-olds’ (χ^2^ = 19.45, df = 2, p < .001) and five-year-olds’ (χ^2^ = 68.60, df = 2, p < .001) animacy judgments about humanoid robots. Four-year-olds’ (B = .56, p < .05) and five-year-olds’ (B = 1.37, p < .001) psychological property projections were found to have a significant effect on the prediction of their animacy judgments. With the score for psychological property projection increasing by one point, the likelihood of judging that the robot as alive rose 1.75 times for four-year-olds and 3.94 times for five-year-olds.

These findings suggest that—depending on age—children’s biological and psychological property projections had different impacts on animacy judgments. Psychological property projection was a factor affecting the animacy judgment in four- and five-year-olds. However, this result cannot be interpreted that they did not consider biological properties when judging an object’s animacy. The findings about children’s property projections in an earlier section demonstrated that children in this age bracket did not tend to attribute biological properties to robots. Therefore, it is possible that their biological property projections did not have a statistically significant effect on their animacy judgments. Inferring whether robots possess psychological properties appeared to be a more significant factor influencing four- and five-year-olds’ animacy judgments.

## Discussion

In this study, we examined children’s biological concepts especially the distinction between living and nonliving kinds using humanoid robots. The growing evidence about children’s ability to differentiate living from nonliving objects are in contrast to Piaget’s early ideas [6] about children’s thought processes. However, it is premature to conclude that young children can distinguish living and nonliving kinds. Most previous research included items that were typical and clearly classifiable as living or nonliving. Therefore, it is difficult to state that these studies reflected the diversity of children’s real worlds. For this reason, we investigated children’s understanding about potentially confusing objects like robots and inquired how firm children’s ontological distinctions were when asked to classify objects with ambiguous category memberships. By experimentally manipulating robot attributes, we determined the features relevant to children of different ages for the construction of animacy concepts. Focusing on attributes that were hypothesized to influence children’s category judgments such as robots’ autonomous movement and their contingent behavior, we examined children’s reasoning about biological and psychological properties to understand their animacy concepts. Previous studies reviewed above suggested that children do not distinguish uniformly when reasoning about boundary objects (e.g., [17]). Instead, they seemed to judge, depending on whether they were asked about life status, biological characteristics, or psychological features. Finally, we investigated whether children's biological and psychological property reasoning affected their animacy judgments. From the data in this study, the following conclusions were drawn.

First, there were developmental differences in children's animacy judgments about boundary objects. Since robots are new agents for children, they should use features of these novel objects to judge their life status. Three-year-olds assessed the boundary objects as anthropomorphic creatures, regardless of their properties. Four-year-olds considered mobility as an important cue to animacy judgments and five-year-olds reacted sensitively to robots’ psychological features.

The nativists (e.g., [1]) asserted that children are instantaneously judging all objects in the world through innate biological theories. However—depending on their features—the young subjects in this study displayed different understandings on the boundary objects. Moreover, Wellman and Gelman pointed out that, in order to acknowledge the identity of a biological domain, children should distinguish between living and nonliving by using consistent principles [33]. From this viewpoint, children’s different judgments on robots suggest that it is as of yet unclear whether there is a biological domain applicable to all the objects in the world. Inagaki and Hatano, proponents of children’s naïve biology, argued that children explain life phenomena based on “vital causality,” a mechanism specific to the biology domain [34]. However, it is difficult to explain children’s animacy judgments of boundary object like robots by applying naïve biology.

The constructivist's claim (e.g., [6, 7]) seems to fit the results of children’s animacy judgments in this study better. It is argued in constructivism that preoperational children have not yet mastered scientific and logically complete biological concepts. It is claimed that children’s biological concepts continue to develop through acquired experience and learning. In this study, it could not be concluded whether children are born with biological theories. What we can say is that children may possess a naïve biology as the nativists claim, but there seems to be room to change by learning.

Second, children’s animacy judgments about boundary objects depend on the object features. Although the grounds for judging the animacy used by three-year-olds are unclear, their older counterparts appraised animacy based on the objects’ biological or psychological features. This can corroborate earlier study results on the continuous growth of children’s biological concepts from age 3 to 5, and age 4 as a turning point in development [22]. In addition, the present study confirmed the possibility that four- and five-year-olds judge animacy in a multifaceted way and not based on a single standard of biological features.

The fact that five-year-olds took psychological features into account when judging the animacy of boundary objects may be related to the child's ability of understanding the mind. Previous studies suggested that children understood objects as agents with minds possessing intentions, desires, and beliefs when they perceived that the object has a face or eyes (e.g., [27, 31, 32]), that it interacts with others contingently (e.g., [27, 29, 35]), or that it moves autonomously (e.g., [27–29]). In this study, humanoid robots, which are novel agents for children and accurately imitated human beings, were used as a research tool, unlike previous investigations with a stuffed orangutan [27] or a mechanical pincer [36]. The humanoid robots had mentalistic cues that allowed children to think of them as mindful agents, specifically, since they had faces and eyes (R1, R2, R3, R4), moved spontaneously (R3, R4), and behaved contingently (R2, R4). For this reason, three-year-olds seemed to have judged all robot types as animate. On the other hand, five-year-olds appeared to be more influenced by the contingency of the robots than their appearance or movements. It is possible that a child who perceived robots as agents with minds assessed them as alive [37, 38].

There were developmental differences in children’s reasoning about boundary objects in addition to the different biological and psychological property projections. Older children were less likely to attribute biological properties to boundary objects than younger ones. These developmental differences showed qualitative changes from the age of 4. Three-year-olds tended to over-generalize their understanding of humans to that of humanoid robots because they were greatly affected by the perceptual similarity between the two. However, older children did not attribute biological properties to robots since they perceived these boundary objects as artifacts.

Three-year-olds responded consistently to the property projection questions about the boundary objects. They believed that the robots were alive and coherently attributed biological and psychological properties to them. To interpret this result as children's innate biological “theory,” it is necessary to determine the objects’ animate properties with consistency, even if age increases. However, four- and five-year-olds who supposedly have more mature biological theories than three-year-olds attributed biological and psychological properties about the boundary objects differently. In other words, they inferred that robots did not have biological properties, but psychological ones. The children’s reasoning of psychological properties led them to judge robots as living.

It is important to note here that the “vital causality” presented by Inagaki and Hatano [5] as evidence for the existence of naïve biology did not apply to the children’s reasoning about the robots in this study. They clearly denied that robots have a series of life mechanisms, such as eating, growing, breathing, and biological origins, but at the same time judged the robots as living. The psychological features of the robots influenced children’s animacy judgments. One possibility of older children judging the boundary objects as living entities lies in their reasoning that objects with psychological properties also possess biological properties. However, one needs to be careful in explaining that children’s biological theories are the same scientific “theories” as those of adults [39]. Although the children attributed psychological properties to robots, they apparently denied biological properties to the object at the same time.

These beliefs may be the result of learning about certain animate properties. For example, it is possible that children did not attribute a “growing” property to robots because they had learned that developing things need soft exteriors rather than metal or plastic shells [19]. However, knowledge of this selective fact did not seem to prevent children from believing that the robots can actually experience emotions and think like humans. Thus, as in the constructivist’s view, children’s existing beliefs about boundary objects seemed to change through acquired experience and learning. The existence of scientific biological theories of children, as claimed by the nativists, appears only to be meaningful for biological property projections. Even five-year-olds’ understanding of animacy about objects (at least boundary objects) was affected by the objects’ features although their biological concepts are considered stable. This suggests that the biological concepts of children at the preoperational stage are not yet completed but continue to develop.

Specifically, the findings that the influence of biological and psychological property projections differed in children’s animacy judgments about the boundary objects suggest that preoperational children may not yet be able to organically link the relationship between concepts of aliveness, biological properties, and psychological characteristics. It is also conceivable that children’s understanding of animacy is in a flexible state, rather than a firm structure. They seem to reconstruct the concept of boundary objects by acquiring knowledge and to learn about novel objects by excluding certain biological properties from them. The naïve biology theorists’ claims appear reasonable when explaining children’s animacy judgments about typical living and nonliving entities. However, when children were asked to reason about objects whose category boundaries are ambiguous, they seemed to distinguish between living and nonliving kinds on grounds other than the “vital causality” that has been suggested by the naïve biology theorists. The child's innate theory of biology may possibly not be universal or unchangeable, but exist in the form of a skeletal and flexible framework [4].

Although this study provided insights into the development of children’s biological concepts by exploring their understanding of life phenomena about boundary objects, there were also limitations that should be addressed. First, we examined children’s understanding about humanoid robots using video demonstrations of human-robot interactions because this allowed providing each child with the same experience. This would not have been possible with real child-robot interactions. However, the results of children actually interacting with robots may be different from observing the robots. Previous studies suggested that even young children might be sensitive to subtle features like movement and contingency in making life judgment after interaction with objects (e.g., [40, 41]). Hence, the experience of interacting directly with the robots may change the children’s form of reasoning and it is necessary to take this into account in future studies with actual child-robot interactions. Second, we conducted our experiment with a robot that has speech function. However, the verbal responsiveness itself may affect children’s understanding of robot. In a follow-up study, it will be necessary to further investigate the children’s responses to the verbal and nonverbal robots in the contingent and non-contingent conditions.

In conclusion, children may have a rudimentary ability to distinguish between living and nonliving objects. However, the way in which these abilities are integrated into biological concepts seems to be better explained by constructivism. Preoperational children’s understanding of animacy does not seem to begin with a consistent and logical theory, but it rather appears to be a consequence of experience, learning, constant change, and development by the acquisition of piecemeal knowledge. If we acknowledge that all parts of the biological theory can be modified [2] and that its core contents are changeable [39], the dichotomous biological theory of traditional living and nonliving kinds can also change with the advancement of science. New technologies like robotics have emerged and children may perceive boundary objects as belonging to a new category with both animate and inanimate properties. Previous studies (e.g., [17, 37, 42–44]) demonstrated that people understood robots as something between living and nonliving. Children's understanding of boundary objects such as a humanoid robot is too limited for a description by the traditional theory of biological concepts. A new approach may be needed [45] in this context of theory construction.

## Supporting information

S1 Table Difference in animacy judgment for each type of robot by children’s age. (DOCX)

S2 Table Biological property projections scores according to children’s age. (DOCX)

S3 Table Difference in biological property projections scores according to age and robot types. (DOCX)

S4 Table Biological property projection of children who responded that robots are alive: Number (percentage) of “yes” responses in the biological property questions. (DOCX)

S5 Table Psychological property projections scores according to children’s age. (DOCX)

S6 Table Difference in psychological property projections scores according to age and robot types. (DOCX)

S7 Table Differences of psychological property projections scores according to age and robot types. (DOCX)

S8 Table Psychological property projections of children who responded that robots are alive: Number (percentage) of “yes” responses in the psychological property questions. (DOCX)

S9 Table Results of binary logistic regression analysis for the relative influence of children’s property projections on animacy judgments about humanoid robots. (DOCX)

S1 File Interview questions for children. (DOCX)

S2 File Parent questionnaire (English). (PDF)

S3 File Parent questionnaire (Korean). (PDF)
