# Supplementary material for: Between living and nonliving: Young children’s animacy judgments and reasoning about humanoid robots
Source: PLoS One. 2019 Jun 28;14(6):e0216869. doi: 10.1371/journal.pone.0216869 (PMC6599145; doi:10.1371/journal.pone.0216869)
Supplement: S1 Table — (DOCX) [file pone.0216869.s001.docx]

**S2 Table. Biological property projections scores according to children’s age**

| Age | Type of robot | | | | *Mean* |
| --- | --- | --- | --- | --- | --- |
|  | R1 | R2 | R3 | R4 |  |
|  | *M(SD)* | *M(SD)* | *M(SD)* | *M(SD)* |  |
| 3-yr-olds  *(n=40)* | 2.37(1.41) | 2.62(1.19) | 2.62(1.31) | 3.03(1.23) | 2.66(.16) |
| 4-yr-olds  *(n=40)* | .83(1.30) | .95(1.43) | .80(1.22) | 1.27(1.55) | .96(.16) |
| 5-yr-olds  *(n=40)* | .15(.48) | .27(.82) | .20(.79) | .55(1.13) | .29(.16) |
| Total (N=120) | 1.12(1.47) | 1.28(1.53) | 1.21(1.53) | 1.62(1.67) |  |

▪ R1 = “immobile & non-contingent”, R2 = “immobile & contingent”, R3 = “mobile & non-contingent”, R4 = “mobile & contingent”
