# Supplementary material for: Between living and nonliving: Young children’s animacy judgments and reasoning about humanoid robots
Source: PLoS One. 2019 Jun 28;14(6):e0216869. doi: 10.1371/journal.pone.0216869 (PMC6599145; doi:10.1371/journal.pone.0216869)
Supplement: S3 Table — (DOCX) [file pone.0216869.s003.docx]

**S3 Table. Difference in biological property projections scores according to age and robot types**

| Source |  | *SS* | *df* | *MS* | *F* | *Partial* η ^2^ |
| --- | --- | --- | --- | --- | --- | --- |
| Between-subjects | Age | 477.24 | 2 | 238.62 | 56.92^***^ | .493 |
|  | Error | 490.49 | 117 | 4.19 |  |  |
| Within-subjects | Type of robot | 17.09 | 3 | 6.71 | 11.36^***^ | .088 |
|  | Age×Type of robot | 1.13 | 6 | .22 | .38 | .006 |
|  | Error | 176.03 | 298 | .59 |  |  |

*^***^p<*.001
