# Supplementary material for: Between living and nonliving: Young children’s animacy judgments and reasoning about humanoid robots
Source: PLoS One. 2019 Jun 28;14(6):e0216869. doi: 10.1371/journal.pone.0216869 (PMC6599145; doi:10.1371/journal.pone.0216869)
Supplement: S4 Table — (DOCX) [file pone.0216869.s004.docx]

**S4 Table. Biological property projection of children who responded that robots are alive: Number (percentage) of “yes” responses in the biological property questions**

| Type of robot | Age | Biological property projection | | | |
| --- | --- | --- | --- | --- | --- |
|  |  | Eating | Growing | Breathing | Biological origin |
| R1 | 3-yr-olds *(n = 34)* | 25 (73.5) | 29 (85.3) | 24 (70.6) | 13 (38.2) |
|  | 4-yr-olds *(n = 25)* | 11 (44.0) | 8 (32.0) | 9 (36.0) | 2 (8.0) |
|  | 5-yr-olds *(n = 14*) | 3 (21.4) | 0 (0.0) | 2 (14.3) | 1 (7.1) |
|  | Total (N = 73) | 39 (53.4) | 37 (50.7) | 35 (47.9) | 16 (21.9) |
|  | *χ²(df)* | 12.18 (2)^**^ | 34.17 (2)^***^ | 14.77 (2)^**^ | 9.91 (2)^**^ |
| R2 | 3-yr-olds *(n = 37)* | 27 (73.0) | 32 (86.5) | 33 (89.2) | 12 (32.4) |
|  | 4-yr-olds *(n = 26)* | 11 (42.3) | 10 (38.5) | 9 (34.6) | 4 (15.4) |
|  | 5-yr-olds *(n = 24)* | 3 (12.5) | 2 (8.3) | 4 (16.7) | 2 (8.3) |
|  | Total (N = 87) | 41 (47.1) | 44 (50.6) | 46 (52.9) | 18 (20.7) |
|  | *χ²(df)* | 22.56 (2)^***^ | 38.73 (2)^***^ | 36.65 (2)^***^ | 3.27 (2) |
| R3 | 3-yr-olds *(n = 35)* | 26 (74.3) | 29 (82.9) | 25 (71.4) | 16 (45.7) |
|  | 4-yr-olds (*n = 30)* | 10 (33.3) | 9 (30.0) | 10 (33.3) | 1 (3.3) |
|  | 5-yr-olds *(n = 19)* | 2 (10.5) | 2 (10.5) | 3 (15.8) | 1 (5.3) |
|  | Total (N = 84) | 38 (45.2) | 40 (47.6) | 38 (45.2) | 18 (21.4) |
|  | *χ²(df)* | 22.88 (2)^***^ | 31.64 (2)^***^ | 18.06 (2)^***^ | 21.04 (2)^***^ |
| R4 | 3-yr-olds *(n = 38)* | 30 (78.9) | 33 (86.8) | 32 (84.2) | 20 (52.6) |
|  | 4-yr-olds *(n = 34)* | 16 (47.1) | 13 (38.2) | 15 (44.1) | 6 (17.6) |
|  | 5-yr-olds *(n = 29)* | 3 (10.3) | 5 (17.2) | 7 (24.1) | 3 (10.3) |
|  | Total (N = 101) | 49 (48.5) | 51 (50.5) | 54 (53.5) | 29 (28.7) |
|  | *χ²(df)* | 31.03 (2)^***^ | 34.96 (2)^***^ | 25.66 (2)^***^ | 17.44 (2)^***^ |

*^**^p* < .01, *^***^p* < .001

▪ R1 = “immobile & non-contingent”, R2 = “immobile & contingent”, R3 = “mobile & non-contingent”, R4 = “mobile & contingent”
