# Supplementary material for: Between living and nonliving: Young children’s animacy judgments and reasoning about humanoid robots
Source: PLoS One. 2019 Jun 28;14(6):e0216869. doi: 10.1371/journal.pone.0216869 (PMC6599145; doi:10.1371/journal.pone.0216869)
Supplement: S5 Table — (DOCX) [file pone.0216869.s005.docx]

**S5 Table. Psychological property projections scores according to children’s age**

| Age | Type of robot | | | | *Mean* |
| --- | --- | --- | --- | --- | --- |
|  | R1 | R2 | R3 | R4 |  |
|  | *M(SD)* | *M(SD)* | *M(SD)* | *M(SD)* |  |
| 3-yr-olds  *(n=40)* | 1.55(.75) | 1.65(.66) | 1.62(.74) | 1.82(.50) | 1.66(.10) |
| 4-yr-olds  *(n=40)* | .85(.92) | 1.25(.84) | .95(.93) | 1.50(.72) | 1.14(.10) |
| 5-yr-olds  *(n=40)* | .52(.75) | 1.37(.74) | .65(.80) | 1.48(.75) | 1.01(.10) |
| Total (n=120) | .98(.91) | 1.43(.76) | 1.08(.92) | 1.60(.68) |  |

▪ R1 = “immobile & non-contingent”, R2 = “immobile & contingent”, R3 = “mobile & non-contingent”, R4 = “mobile & contingent”
