# Supplementary material for: Between living and nonliving: Young children’s animacy judgments and reasoning about humanoid robots
Source: PLoS One. 2019 Jun 28;14(6):e0216869. doi: 10.1371/journal.pone.0216869 (PMC6599145; doi:10.1371/journal.pone.0216869)
Supplement: S6 Table — (DOCX) [file pone.0216869.s006.docx]

**S6 Table. Difference in psychological property projections scores according to age and robot types**

| Source |  | *SS* | *df* | *MS* | *F* | *Partial* η ^2^ |
| --- | --- | --- | --- | --- | --- | --- |
| Between-subjects | Age | 9.65 | 2 | 4.82 | 12.90^***^ | .181 |
|  | Error | 43.75 | 117 | .37 |  |  |
| Within-subjects | Type of robot | 30.96 | 3 | 11.20 | 36.19^***^ | .236 |
|  | Age×Type of robot | 9.71 | 6 | 1.76 | 5.68^***^ | .088 |
|  | Error | 100.08 | 323 | .31 |  |  |

*^***^p*<.001
