# Supplementary material for: Between living and nonliving: Young children’s animacy judgments and reasoning about humanoid robots
Source: PLoS One. 2019 Jun 28;14(6):e0216869. doi: 10.1371/journal.pone.0216869 (PMC6599145; doi:10.1371/journal.pone.0216869)
Supplement: S7 Table — (DOCX) [file pone.0216869.s007.docx]

**S7 Table. Differences of psychological property projections scores according to age and robot types**

| Age | Type of robot | M(SD) | *SS* | *df* | *MS* | *F* |
| --- | --- | --- | --- | --- | --- | --- |
| 3-yr-olds | R1 | 1.55(.75)^a^ | 1.63 | 3 | .54 | 1.21 |
|  | R2 | 1.65(.66)^a^ |  |  |  |  |
|  | R3 | 1.63(.74)^a^ |  |  |  |  |
|  | R4 | 1.83(.50)^a^ |  |  |  |  |
| 4-yr-olds | R1 | .85(.92)^ab^ | 10.48 | 3 | 3.49 | 4.76*^**^* |
|  | R2 | 1.25(.84)^bd^ |  |  |  |  |
|  | R3 | .95(.93)^bc^ |  |  |  |  |
|  | R4 | 1.50(.72)^d^ |  |  |  |  |
| 5-yr-olds | R1 | .52(.75)^ac^ | 28.57 | 3 | 9.52 | 16.43*^***^* |
|  | R2 | 1.37(.74)^b^ |  |  |  |  |
|  | R3 | .65(.80)^ac^ |  |  |  |  |
|  | R4 | 1.48(.75)^b^ |  |  |  |  |
| Type of robot | Age | M(SD) | *SS* | *df* | *MS* | *F* |
| R1 | 3-yr-olds | 1.55(.75)^a^ | 21.95 | 2 | 10.98 | 16.68*^***^* |
|  | 4-yr-olds | .85(.92)^b^ |  |  |  |  |
|  | 5-yr-old | .52(.75)^b^ |  |  |  |  |
| R2 | 3-yr-olds | 1.65(.66)^a^ | 3.35 | 2 | 1.68 | 2.97 |
|  | 4-yr-olds | 1.25(.84)^a^ |  |  |  |  |
|  | 5-yr-old | 1.37(.74)^a^ |  |  |  |  |
| R3 | 3-yr-olds | 1.63(.74)^a^ | 19.95 | 2 | 9.98 | 14.52*^***^* |
|  | 4-yr-olds | .95(.93)^b^ |  |  |  |  |
|  | 5-yr-old | .65(.80)^b^ |  |  |  |  |
| R4 | 3-yr-olds | 1.83(.50)^a^ | 3.05 | 2 | 1.53 | 3.45 |
|  | 4-yr-olds | 1.50(.72)^a^ |  |  |  |  |
|  | 5-yr-old | 1.48(.75)^a^ |  |  |  |  |

*^**^p*<.01, *^***^p*<.001

*Note:* The alphabet superscript letters (^a-d^) indicate which values are significantly different using Scheffé’s test. Values that are not significantly different based on the post-hoc Scheffé contrast have the common superscripts. For example, in 4-yr-old’s group, the score for R1 would mean that it is significantly different from the score for R4 because they do not have any common superscript letter. On the other hand, the score for R1 does not differ from both scores for R2 and R3 because all of them have a common superscript letter “b”.

▪ R1 = “immobile & non-contingent”, R2 = “immobile & contingent”, R3 = “mobile & non-contingent”, R4 = “mobile & contingent”
