# Supplementary material for: Between living and nonliving: Young children’s animacy judgments and reasoning about humanoid robots
Source: PLoS One. 2019 Jun 28;14(6):e0216869. doi: 10.1371/journal.pone.0216869 (PMC6599145; doi:10.1371/journal.pone.0216869)
Supplement: S8 Table — (DOCX) [file pone.0216869.s008.docx]

**S8 Table. Psychological property projections of children who responded that robots are alive: Number (percentage) of “yes” responses in the psychological property questions**

| Type of robot | Age | Psychological property projection | |
| --- | --- | --- | --- |
|  |  | Emotion | Thinking |
| R1 | 3-yr-olds (n = 34) | 30 (88.2) | 27 (79.4) |
|  | 4-yr-olds (n = 25) | 13 (52.0) | 13 (52.0) |
|  | 5-yr-olds (n = 14) | 8 (57.1) | 9 (64.3) |
|  | Total (N = 73) | 51 (69.9) | 49 (67.1) |
|  | *χ²(df)* | 10.32 (2)^**^ | 4.97 (2) |
| R2 | 3-yr-olds (n = 37) | 33 (89.2) | 30 (81.1) |
|  | 4-yr-olds (n = 26) | 20 (76.9) | 15 (57.7) |
|  | 5-yr-olds (n = 24) | 24 (100.0) | 18 (75.0) |
|  | Total (N = 87) | 77 (88.5) | 63 (72.4) |
|  | *χ²(df)* | 4.41 (2) | 4.11 (2) |
| R3 | 3-yr-olds (n = 35) | 31 (88.6) | 30 (85.7) |
|  | 4-yr-olds (n = 30) | 18 (60.0) | 16 (53.3) |
|  | 5-yr-olds (n = 19) | 9 (47.4) | 9 (47.4) |
|  | Total (N = 84) | 58 (69.0) | 55 (65.5) |
|  | *χ²(df)* | 11.57 (2)^**^ | 11.06 (2)^**^ |
| R4 | 3-yr-olds (n = 38) | 36 (94.7) | 33 (86.8) |
|  | 4-yr-olds (n = 34) | 30 (88.2) | 26 (76.5) |
|  | 5-yr-olds (n = 29) | 27 (93.1) | 23 (79.3) |
|  | Total (N = 101) | 93 (92.1) | 82 (81.2) |
|  | *χ²(df)* | 1.10 (2) | 1.36 (2) |

*^**^p* < .01, *^***^p* < .001

▪ R1 = “immobile & non-contingent”, R2 = “immobile & contingent”, R3 = “mobile & non-contingent”, R4 = “mobile & contingent”
