# Supplementary material for: Between living and nonliving: Young children’s animacy judgments and reasoning about humanoid robots
Source: PLoS One. 2019 Jun 28;14(6):e0216869. doi: 10.1371/journal.pone.0216869 (PMC6599145; doi:10.1371/journal.pone.0216869)
Supplement: S9 Table — (DOCX) [file pone.0216869.s009.docx]

**S9 Table. Results of binary logistic regression analysis for the relative influence of children’s property projections on animacy judgments about humanoid robots**

| Age | Variables | *B* | Wald *χ²* | Exp(*B*) | 95% CI |
| --- | --- | --- | --- | --- | --- |
| 3-yr-olds | Biological property projection | .50^*^ | 4.88 | 1.65 | 1.06-2.58 |
|  | Psychological property projection | .58 | 2.38 | 1.78 | .86-3.69 |
|  | Overall correct prediction (%) | 89.4% | | | |
|  | -2 Log Likelihood | 91.73 | | | |
|  | Model *χ²* | 16.62^***^ | | | |
| 4-yr-olds | Biological property projection | .35 | 2.92 | 1.43 | .95-2.14 |
|  | Psychological property projection | .56^*^ | 5.04 | 1.75 | 1.07-2.86 |
|  | Overall correct prediction (%) | 71.9% | | | |
|  | -2 Log Likelihood | 170.68 | | | |
|  | Model *χ²* | 19.45^***^ | | | |
| 5-yr-olds | Biological property projection | 18.05 | .00 | 6.89 | .00-.00 |
|  | Psychological property projection | 1.37^***^ | 29.50 | 3.94 | 2.40-6.46 |
|  | Overall correct prediction (%) | 76.3% | | | |
|  | -2 Log Likelihood | 152.30 | | | |
|  | Model *χ²* | 68.60^***^ | | | |
| Total | Biological property projection | .56^***^ | 21.46 | 1.75 | 1.38-2.22 |
|  | Psychological property projection | .87^***^ | 34.00 | 2.39 | 1.78-3.21 |
|  | Overall correct prediction (%) | 77.7% | | | |
|  | -2 Log Likelihood | 439.46 | | | |
|  | Model *χ²* | 132.77^***^ | | | |

^*^*p* < .05, ^***^*p* < .001
