# Supplementary material for: Between living and nonliving: Young children’s animacy judgments and reasoning about humanoid robots
Source: PLoS One. 2019 Jun 28;14(6):e0216869. doi: 10.1371/journal.pone.0216869 (PMC6599145; doi:10.1371/journal.pone.0216869)
Supplement: S1 File — (DOCX) [file pone.0216869.s010.docx]

**Interview questions for children’s animacy judgment and biological/psychological property projection**

1. **Korean version**

| ID |  | | | | |
| --- | --- | --- | --- | --- | --- |
| 이름 |  | | | | |
| 연령 |  | | | | |
| 생년월일 |  | | | | |
| 질의응답 | | | | | |
|  | | R1 | R2 | R3 | R4 |
| 이것은 살아있는 것이에요 안 살아있는 것이에요? | |  |  |  |  |
| 이것은 물이나 음식을 진짜로 먹을까? | |  |  |  |  |
| 이것은 키가 쑥쑥 자랄까? | |  |  |  |  |
| 이것은 숨을 쉴까? | |  |  |  |  |
| 이것은 이것 엄마가 낳아서 태어났을까 사람들이 만들어서 생겨났을까? | |  |  |  |  |
| 이것은 기쁘거나 슬플 때가 있을까? | |  |  |  |  |
| 이것은 생각할 수 있을까? | |  |  |  |  |

1. **English version**

| ID |  | | | | |
| --- | --- | --- | --- | --- | --- |
| Name |  | | | | |
| Age |  | | | | |
| Date of birth |  | | | | |
| Questions/Answers | | | | | |
|  | | R1 | R2 | R3 | R4 |
| Is it alive or not alive? | |  |  |  |  |
| Does it need water or food? | |  |  |  |  |
| Does it grow? | |  |  |  |  |
| Does it breathe? | |  |  |  |  |
| Was it born or was it made by man? | |  |  |  |  |
| Can this one feel happy or unhappy? | |  |  |  |  |
| Can this one think? | |  |  |  |  |
