# Supplementary material for: Between living and nonliving: Young children’s animacy judgments and reasoning about humanoid robots
Source: PLoS One. 2019 Jun 28;14(6):e0216869. doi: 10.1371/journal.pone.0216869 (PMC6599145; doi:10.1371/journal.pone.0216869)
Supplement: S2 File — (PDF) [file pone.0216869.s011.pdf]

ID | | | | |

Parent questionnaire

Dear parent

We sincerely thank you for participating in the study. The following questions are intended to confirm the general characteristics of the child who participate in the study and the child’s prior experience of science and technology related to robots. The information you provide is for research purposes only. In addition, the information is statistically processed to ensure complete privacy. I would like to ask you to answer every questions.

We sincerely appreciate your cooperation. Once you have completed your questionnaire, please return it to the research director.

※ If you have any questions about the questionnaire, please contact:
Department of Child and Family Studies, SNU, Min-Kyung Kim
Email: mkkim82@snu.ac.kr

Ⅰ. Here are some general questions about you and your child. Please read each question and mark √.

1. What is your child's gender?
① male ( ) ② female ( )

2. What is your child's birth order?
① first ( ) ② second ( ) ③ third or more ( )

3. How many siblings does your child have?
① none ( ) ② one ( ) ③ more than two ( )

4. How long has your child spent in day care center or kindergarten (including the time (s)he has been in the past)?
① less than 6 months ( )
② more than 6 months ~ less than 1 year ( )
③ more than 1 year ~ 2 years ( )
④ more than 2 years ~ 3 years ( )
⑤ more than 3 years ( )

5. This is a question about the educational attainment of child's parents. Please indicate the final educational background of you and your spouse. (Check only if applicable)
(1) Mother: ① middle school graduate ( ), ② high school graduate ( ), ③ college graduate or university dropout ( ), ④ university graduate ( ), ⑤ graduate school ( )
(2) Father: ① middle school graduate ( ), ② high school graduate ( ), ③ college graduate or university dropout ( ), ④ university graduate ( ), ⑤ graduate school ( )

6. This is a question about parents' job. Please indicate job of you and your spouse. If there is no suitable choice, please write directly in the other field. (Check only if applicable)

job	mother	father
① professional job		
② office manager		
③ sales service		
④ production worker		
⑤ housewife		
⑥ inoccupation		
⑦ other (Please specify if it does not correspond to 1 ~ 6)		

7. What is your monthly average household income?
① less than 2 million won ( ) ② more than 2 million won ~ 3 million won ( )
③ more than 3 million won ~ 4 million won ( ) ④ more than 4 million won ~ 5 million won ( )
⑤ more than 5 million won ( )

Ⅱ. The following questions are related to the robot experience of the child. Please read each question and mark √.

1. Do you have a robot in your home (such as a robotic vacuum cleaner) or a robotic toy (such as a ‘또봇’)?
① Yes (　　)　　　　② No (　　)

2. Has your child ever used an educational robot like the one pictured below?
① Yes (　　)　　　　② No (　　)

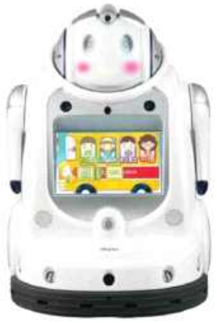
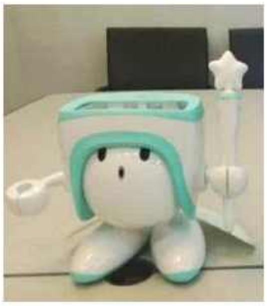
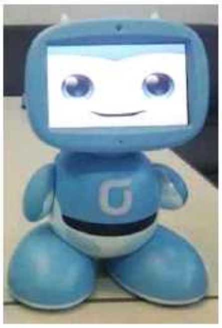

3. Have your child ever watched videos related to robots (eg, Wall-E)?
① Yes (　　)　　　　② No (　　)

4. Has your child assembled or created robots by herself/himself (eg, Lego Mindstorm)?
① Yes (　　)　　　　② No (　　)

5. Have your child ever seen an intelligent robot (a humanoid intelligent robot / humanoid robot) that looks like someone who can actually interact with humans, as shown in the picture below?
① Yes (　　)　　　　② No (　　)

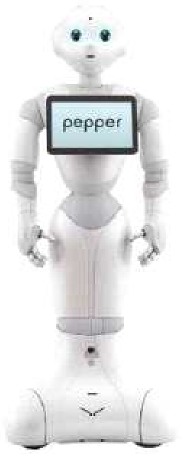

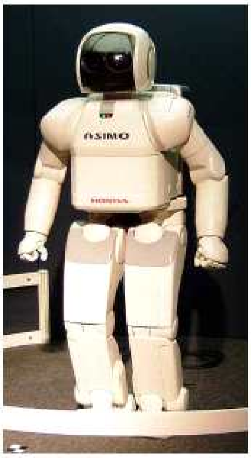

6. Have you ever seen an intelligent robot (an animal intelligent robot) that looks like an animal that can actually interact with humans, such as the one pictured below?

① Yes (   )   ② No (   )

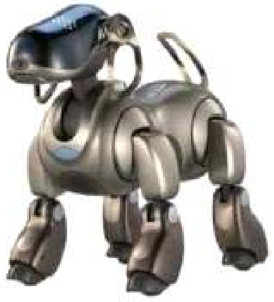

7. How well does your child understand how robots work (eg, understanding the mechanical structure inside the robot, the principle of how the robot behaves or makes sounds, etc.)?

(s)he has no idea.	(s)he does not know well.	(s)he knows a little.	(s)he knows very well.

※ Thank you very much for your reply.
