# Supplementary material for: Between living and nonliving: Young children’s animacy judgments and reasoning about humanoid robots
Source: PLoS One. 2019 Jun 28;14(6):e0216869. doi: 10.1371/journal.pone.0216869 (PMC6599145; doi:10.1371/journal.pone.0216869)
Supplement: S3 File — (PDF) [file pone.0216869.s012.pdf]

ID

보호자용 질문지

안녕하십니까?

유아의 인지 발달 연구에 동의해 주셔서 진심으로 감사드립니다. 다음은 연구대상 유아의 일반적 특징과 로봇과 관련된 과학기술의 사전 경험을 확인하기 위한 내용입니다. 응답하신 내용은 연구 목적으로만 사용되며, 통계 처리되어 개인적인 비밀이 완전히 보장됩니다. 한 문항이라도 응답을 안 하시면 애써 작성하신 질문지 전체를 사용할 수 없게 되므로, 한 문항도 빠짐없이 응답해 주시기를 간곡히 부탁드립니다.

바쁘신 중에도 조사에 협조해 주셔서 진심으로 감사드립니다. 질문지를 완성하신 후에는 연구책임자에게 돌려주시기 바랍니다.

※ 설문지에 관련하여 문의하실 내용이 있으면 다음으로 연락주시기 바랍니다.

문의처 : 서울대학교 아동가족학과 김민경
이메일 : mkkim82@snu.ac.kr

Ⅰ. 다음은 귀하와 연구대상 자녀의 일반적 사항에 대한 질문입니다. 각 문항을 읽고 해당하는 곳에 √표를 해주시기 바랍니다.

1. 귀하의 자녀의 성별은 무엇입니까?
① 남 ( ) ② 여 ( )

2. 귀하의 자녀의 출생순위는 어떻게 됩니까?
① 첫째( ) ② 둘째( ) ③ 셋째 이상( )

3. 귀하의 자녀의 형제 수는 어떻게 됩니까?
① 외동( ) ② 1명( ) ③ 2명 이상( )

4. 귀하의 자녀가 어린이집 또는 유치원(과거에 다녔던 기간 포함)에 다닌 기간은 얼마나 됩니까?
① 6개월 미만(　　)
② 6개월 이상~1년 미만(　　)
③ 1년 이상~2년 미만(　　)
④ 2년 이상~3년 미만(　　)
⑤ 3년 이상(　　)

5. 자녀의 부모님의 학력에 대한 질문입니다. 어머니와 아버지의 최종학력을 표시하여 주십시오. (해당하는 경우에만 체크)
(1) 어머니: ① 중학교 졸업이하(　　), ② 고등학교 졸업(　　), ③ 전문대 졸업 또는 대학교 중퇴(　　),
④ 대학교 졸업(　　),　　⑤ 대학원이상(　　)
(2) 아버지: ① 중학교 졸업이하(　　), ② 고등학교 졸업(　　), ③ 전문대 졸업 또는 대학교 중퇴(　　),
④ 대학교 졸업(　　),　　⑤ 대학원이상(　　)

6. 자녀의 부모님의 직업에 대한 질문입니다. 어머니와 아버님의 직업에 대하여 표시하여 주십시오. 적당한 보기가 없을 경우에는 기타 란에 직접 적어 주십시오. (해당하는 경우에만 체크)

직업	어머니	아버지
① 전문기술직		
② 사무관리직		
③ 판매서비스직		
④ 생산노동직		
⑤ 가정주부		
⑥ 무직		
⑦ 기타(1~6에 해당하지 않는 경우 직접 적어 주세요)		

7. 귀하의 월 평균 가정 소득 수준은 어느 정도입니까?
① 200만원 미만(　　)　　② 200만원 이상 ~ 300만원 미만(　　)
③ 300만원 이상 ~ 400만원 미만(　　) ④ 400만원 이상 ~ 500만원 미만(　　) ⑤ 500만원 이상(　　)

Ⅱ. 다음은 연구 참여 유아의 로봇 경험과 관련된 사항에 대한 질문입니다. 각 문항을 읽고 해당하는 곳에 √표를 해주시기 바랍니다.

1. 귀하의 가정에 **로봇**(예: 로봇청소기 등)**이나 로봇장난감**(예: 장난감 '또봇' 등)이 있습니까?
① 있다 (　　)　　　　　②없다 (　　)

2. 귀하의 자녀는 아래 사진과 같은 **교육용 로봇**을 사용해본 적이 있습니까?
① 있다(　　)　　　　　②없다(　　)

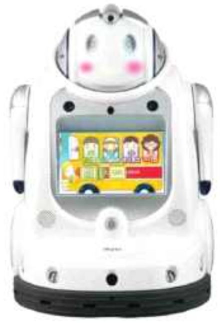
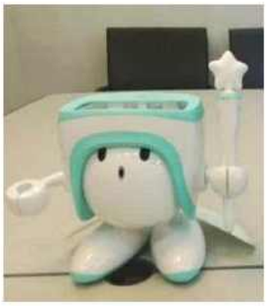
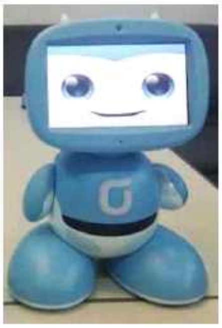

3. 귀하의 자녀는 **로봇과 관련된 동영상**(예: 뽀로로의 '로디', 로보카 폴리, 또봇, 월-E 등)을 시청한 적이 있습니까?
① 있다(　　)　　　　　②없다(　　)

4. 귀하의 자녀는 **로봇을 직접 조립하거나 만들어본 적**이 있습니까(예: 레고 마인드스톰)?
① 있다(　　)　　　　　②없다(　　)

5. 귀하의 자녀는 아래 사진과 같은 실제로 인간과 상호작용이 가능한 **사람 모습을 한 지능형 로봇**(인간형 지능로봇/휴머노이드 로봇)을 본 적이 있습니까?
① 있다 (　　)　　　　　②없다 (　　)

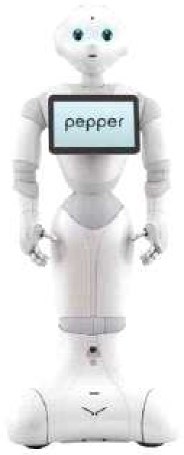

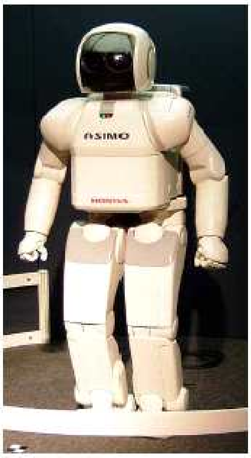

6. 귀하의 자녀는 아래 사진과 같은 실제로 인간과 상호작용이 가능한 **동물 모습을 한 지능형 로봇**(동물형 지능로봇)을 본 적이 있습니까?

① 있다 ( ) ②없다 ( )

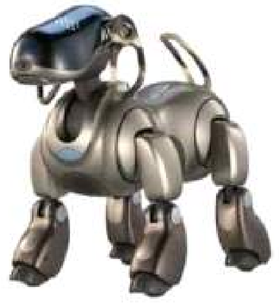

7. 귀하의 자녀는 **로봇의 작동원리**(예: 로봇 내부의 기계적인 구조에 대한 이해, 로봇의 동작이나 음성 등이 나타나는 원리 등)에 대하여 어느 정도 이해하고 있습니까?

전혀 모른다	대체로 모른다	조금 알고 있다	매우 잘 안다

※ 끝까지 응답해 주셔서 대단히 감사합니다.

혹시 빠뜨린 문항이 없으신지 다시 한 번 확인해 주시면 감사하겠습니다.
